# Performance of 2014 NICE defibrillator implantation guidelines in heart failure risk stratification

**DOI:** 10.1136/heartjnl-2015-308939

**Published:** 2016-02-08

**Authors:** Richard M Cubbon, Klaus K Witte, Lorraine C Kearney, John Gierula, Rowenna Byrom, Maria Paton, Anshuman Sengupta, Peysh A Patel, Andrew MN Walker, David A Cairns, Adil Rajwani, Alistair S Hall, Robert J Sapsford, Mark T Kearney

**Affiliations:** 1 Leeds Institute of Cardiovascular and Metabolic Medicine, The University of Leeds, Leeds, UK; 2 Leeds Institute of Clinical Trials Research, The University of Leeds, Leeds, UK; 3 Department of Cardiology, Freeman Hospital, Newcastle upon Tyne, UK; 4 Department of Cardiology, Leeds General Infirmary, Leeds, UK

## Abstract

**Objective:**

Define the real-world performance of recently updated National Institute for Health and Care Excellence guidelines (TA314) on implantable cardioverter-defibrillator (ICD) use in people with chronic heart failure.

**Methods:**

Multicentre prospective cohort study of 1026 patients with stable chronic heart failure, associated with left ventricular ejection fraction (LVEF) ≤45% recruited in cardiology outpatient departments of four UK hospitals. We assessed the capacity of TA314 to identify patients at increased risk of sudden cardiac death (SCD) or appropriate ICD shock.

**Results:**

The overall risk of SCD or appropriate ICD shock was 2.1 events per 100 patient-years (95% CI 1.7 to 2.6). Patients meeting TA314 ICD criteria (31.1%) were 2.5-fold (95% CI 1.6 to 3.9) more likely to suffer SCD or appropriate ICD shock; they were also 1.5-fold (95% CI 1.1 to 2.2) more likely to die from non-cardiovascular causes and 1.6-fold (95% CI 1.1 to 2.3) more likely to die from progressive heart failure. Patients with diabetes not meeting TA314 criteria experienced comparable absolute risk of SCD or appropriate ICD shock to patients without diabetes who met TA314 criteria. Patients with ischaemic cardiomyopathy not meeting TA314 criteria experienced comparable absolute risk of SCD or appropriate ICD shock to patients with non-ischaemic cardiomyopathy who met TA314 criteria.

**Conclusions:**

TA314 can identify patients with reduced LVEF who are at increased relative risk of sudden death. Clinicians should also consider clinical context and the absolute risk of SCD when advising patients about the potential risks and benefits of ICD therapy.

## Introduction

Reduced left ventricular ejection fraction (LVEF) secondary to myocardial infarction is well established as a major risk factor for sudden cardiac death (SCD) due to ventricular arrhythmia.^1^ This observation led to a number of clinical trials examining the effect of prophylactic implantable cardioverter-defibrillators (ICDs) in patients with reduced LVEF due to myocardial infarction.^2–5^ These trials were followed by studies addressing the same question in patients with reduced LVEF associated with heart failure due to ischaemic and non-ischaemic aetiologies.^5^ ^6^

Multiple guidelines now recommend the implantation of ICD as primary prevention in patients with reduced LVEF. The resultant widespread prophylactic implantation of ICD presents an important challenge to healthcare systems as these devices are expensive,^7^ and when implanted,^8^ or activated inappropriately,^9^ are associated with an increased risk of harm to patients. Moreover, some studies of patients at high-risk of SCD (eg, within 1–6 weeks after myocardial infarction) have failed to demonstrate a survival advantage from ICD.^10^ ^11^ ICD use should, therefore, be targeted towards groups of patients most likely to receive clinically meaningful benefit from this treatment.^12^ Indeed, post hoc analysis of the multicenter automatic defibrillator implantation trial (MADIT)-II study has suggested ICD implantation was not associated with benefit in patients at the highest and lowest risk of death, based upon a simple clinical risk score.^13^

The updated UK National Institute for Health and Care Excellence (NICE) technology appraisal (TA314) guidance on device therapy for patients with reduced left ventricular systolic function was published in 2014.^14^ These guidelines, which represent a significant change in UK practice, stratify patients suitable for ICD using New York Heart Association (NYHA) functional class and QRS interval duration from 12-lead ECG. The ability of these guidelines to identify patients at increased risk of SCD has not been examined. In the present report, we used a prospectively recruited unselected cohort of UK patients with heart failure and reduced LVEF to examine the ability of these guidelines to identify patients at increased risk of SCD and test the performance of TA314.

## Methods

This was a multicentre prospective cohort study specifically designed to examine predictors of all-cause mortality and mode of death in patients with heart failure secondary to left ventricular systolic dysfunction.^15^ A total of 1091 patients were recruited between June 2006 and December 2011. All patients were recruited in outpatient clinics located in UK National Health Service hospitals and provided written consent to participate in the study. The study was approved by Leeds West Research Ethics Committee (07/Q1205/17) and conducted in accordance with the principles of the Declaration of Helsinki.

### Eligibility criteria

Stable (no change in clinical status during the previous 3 months) patients over the age of 18 years with signs and symptoms of heart failure and an echocardiographic LVEF of ≤45% were recruited.

### Data collection

At the time of recruitment, a patient case record form detailing clinical and demographic data was completed. The presence of diabetes was defined on the basis of current medication and history taken by recruiting physician. Ischaemic aetiology was determined by the recruiting physician on the basis of detailed history, ECG and clinically indicated imaging, including coronary angiography.^15^ Furosemide dose equivalent was calculated using the ratio 1 mg bumetanide equivalent to 40 mg furosemide. Ramipril and bisoprolol equivalent doses were derived according to our previously published work.^15^ ^16^ NYHA class was defined using standard criteria.^15^ A blood sample was taken for electrolytes, urea, creatinine, liver function and random glucose. Estimated glomerular filtration rate was calculated using the modification of diet in renal disease method.^17^ A 2-dimensional echocardiogram was performed and reported by British Society of Echocardiography (BSE) accredited cardiac physiologists according to BSE guidelines.^18^ A standard 12-lead ECG was performed at the time of recruitment.

### ECG analysis

Standard 12-lead ECGs recorded at 25 mm/s were analysed by two cardiologists blinded to patient characteristics (RMC and AR).

### Application of NICE TA314

All patients with LVEF ≤35%, NYHA class I–III symptoms and QRS duration ≥120 ms were deemed to meet NICE recommendations for ICD implantation (labelled ‘standard TA314’). As TA314 does not clearly stipulate how to define high-risk patients with QRS duration <120 ms, we conducted sensitivity analyses where all patients with LVEF ≤35%, NYHA class I–III symptoms, irrespective of QRS duration, were deemed to meet TA314 criteria (labelled ‘extended TA314’). Patients with permanent pacemakers and no intrinsic QRS interval (n=45) were excluded from analyses, as TA314 offers no guidance on their risk stratification. Patients with missing NYHA class, ECG or echocardiogram data (n=20) were also excluded from analyses, resulting in a study cohort of 1026 patients.

### Mode of death and assessment of ICD shocks

All patients were registered with the UK Office of Population Censuses and Surveys (ONS) to provide details of death including date and location. Mode of death was assessed as previously reported and as follows:^15^ ^16^ (1) sudden death if occurring within 1 h of a change in symptoms or during sleep or while the patient was unobserved *or* appropriate ICD shock, (2) progressive heart failure if death occurred after a documented period of symptomatic or haemodynamic deterioration, (3) other cardiovascular death if not occurring suddenly or in association with progression of heart failure and (4) non-cardiovascular death. For patients with an ICD in situ an appropriate shock was classed as a sudden death event. Each patient with an ICD has a file detailing information at follow-up containing information describing therapies including a copy of ECGs from the device diagnostics of individual episodes. Detection algorithms and therapy settings at the time of therapy are also recorded. Analysis of each episode of device therapy was undertaken by a British Heart Rhythm Society-accredited cardiac physiologist (JG) to assign whether or not therapy was appropriate or inappropriate as previously described.^9^

### Statistics

Descriptive group data are given as mean with SEM. Categorical data are shown as number (%). All analyses were performed using SPSS V.21.0 (IBM, New York, USA). Groups were compared using Student's t test for continuous data and Pearson's χ^2^ for categorical data, using two-sided tests. Survival was compared with log-rank tests. Cox proportional hazards regression analysis was used to define associations between clinical variables and risk of SCD, which are presented as HR. Absolute event rates are provided per 100 patient-years (100 py) of follow-up, with accompanying 95% CIs in parentheses. Statistical significance was accepted at p<0.05.

## Results

### Patient characteristics and mortality

A total of 1026 patients were recruited (characteristics shown in table 1) between June 2006 and December 2011, with mean follow-up of 1360 days (SEM 21), equating to 3823 patient-years of follow-up. The mean age of the cohort was 68 years, and 73.6% were men; 25.7% suffered from diabetes and 63.1% had an underlying ischaemic aetiology. QRS duration ≥120 ms was present in 46.6% (29.4% left bundle branch block (LBBB) morphology, 5.6% right bundle branch block (RBBB) morphology, 11.6% non-specific morphology). Heart failure therapies were used as follows: β-adrenoceptor antagonists in 81%, ACE inhibitors or angiotensin receptor blockers in 89%, mineralocorticoid receptor antagonists in 41%, cardiac resynchronisation therapy in 29% and ICD in 13%. More specifically, 69.2% of patients had no device, 3.3% had ICD alone, 18.3% had cardiac resynchronisation therapy pacemaker (CRT-P) and 9.2% had cardiac resynchronisation therapy defibrillator (CTR-D). All-cause mortality rate was 9 (8.1–10) events/100 py, and SCD rate was 2.1 (1.7–2.6) events/100 py. Of 344 total mortality events, 22.7% were attributable to SCD, 32% to progressive heart failure death, 7.3% to other cardiovascular death, 34.9% to non-cardiovascular death and 3.2% were unclassifiable. Of the 78 SCD events, 28 were appropriate ICD shocks (8 ventricular fibrillation and 20 ventricular tachycardia), with a median programmed threshold for ICD shock of 188 bpm (range 188–240).

**Table 1:** Cohort characteristics

	TA314 does not advise ICD (n=707)	TA314 advises ICD (n=319)	p value
Age (years)	66.9 (0.5)	70.6 (0.6)	<0.001
Heart rate (bpm)	75 (0.8)	74.2 (1)	0.52
Systolic BP (mmHg)	123 (0.8)	116.8 (1.2)	<0.001
QRS interval (ms)	110.6 (1)	152.2 (1.3)	<0.001
Haemoglobin (g/l)	136 (1)	135 (1)	0.38
eGFR (ml/Kg/1.73m2)	56.2 (0.7)	53.2 (0.9)	0.008
LVEDD (mm)	56.2 (0.3)	62.9 (0.5)	<0.001
LVEF (%)	34.7 (0.3)	26.3 (0.4)	<0.001
Ramipril dose (mg/day)	5 (0.1)	4.9 (0.2)	0.73
Bisoprolol dose (mg/day)	3.8 (0.1)	3.3 (0.2)	0.038
Furosemide dose (mg/day)	51.6 (1.9)	57.4 (2.9)	0.094
Diabetes (% (n))	28.6 (202)	19.7 (63)	0.003
Ischaemic aetiology (% (n))	63.6 (450)	59.9 (191)	0.25
CRT (% (n))	14.6 (103)	56.4 (180)	<0.001
ICD (% (n))	9.2 (65)	23.5 (75)	<0.001
NYHA class (% (n))			<0.001
1	22.8 (161)	16.6 (53)	
2	48.4 (342)	41.1 (131)	
3	26.4 (187)	42.3 (135)	
4	2.4 (17)	0	
ACEi / ARB use (% (n))	88.2 (623)	88.6 (280)	0.87
Betablocker use (% (n))	82 (579)	79.7 (252)	0.39
MRA use (% (n))	38.8 (274)	47.5 (150)	0.009

CRT, cardiac resynchronisation therapy; eGFR, estimated glomerular filtration rate; ICD, implantable cardioverter defibrillator; LVEDD, left ventricular end diastolic dimension; LVEF, left ventricular ejection fraction; MRA, mineralocorticoid receptor antagonist; NYHA, New York heart association.

### Performance of NICE TA314 guidelines

**Figure 1:**
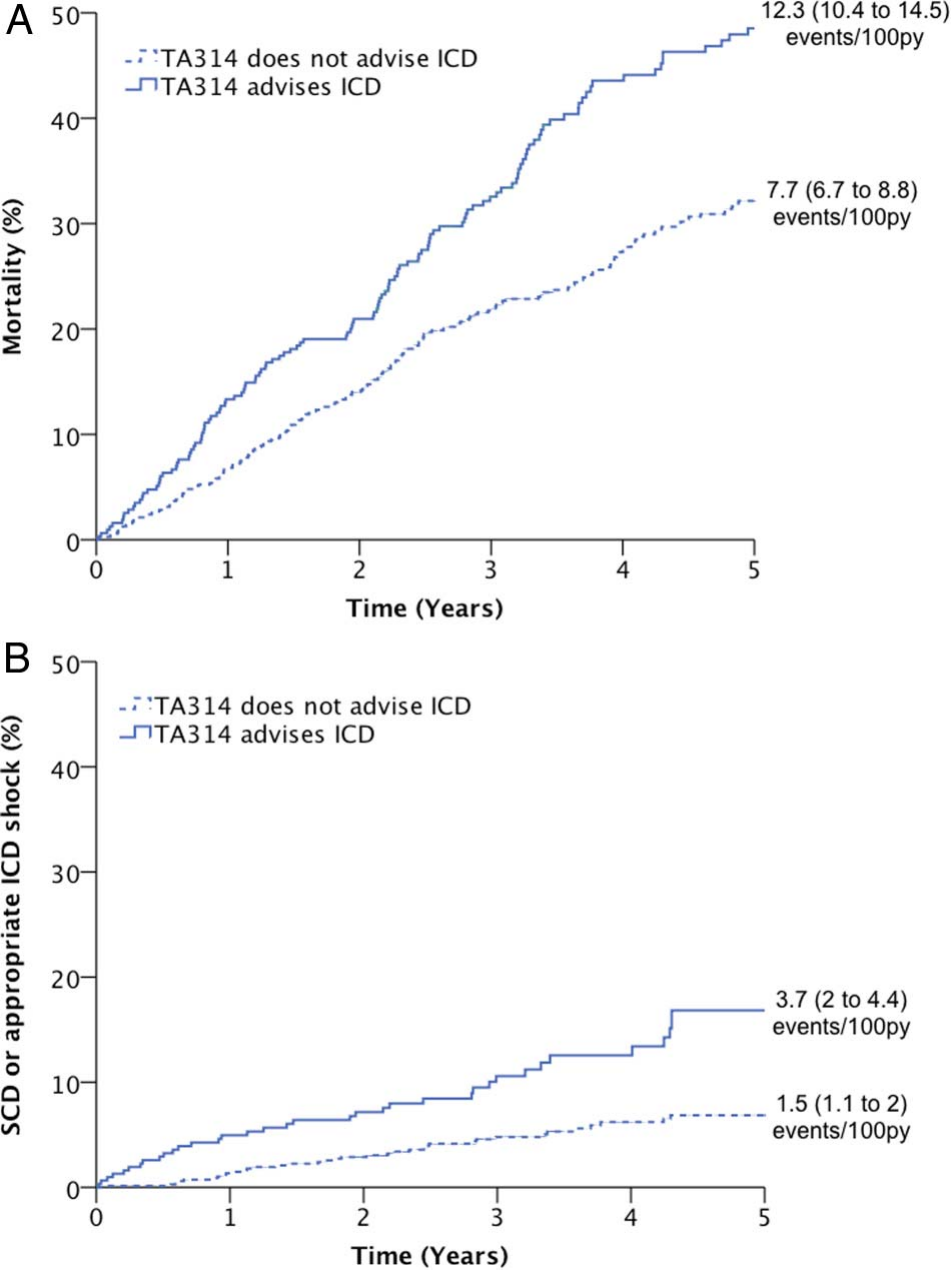
Performance of TA314 in predicting outcome. Kaplan–Meier curves demonstrating that TA314 identifies a subgroup at increased risk of: (A) total mortality and (B) sudden cardiac death (SCD) or appropriate implantable cardioverter-defibrillator (ICD) shock (both comparisons p<0.05).

31.1% of patients fulfilled NICE standard TA314 criteria for ICD implantation. In addition to expected differences in NYHA class and QRS interval, patients recommended to receive ICD were older, had more impaired ventricular systolic function, less often had diabetes, and were much more likely to receive cardiac resynchronisation therapy (table 1). Patients fulfilling TA314 criteria were more likely to die from any cause (HR 1.5 (1.2 to 1.9); p<0.001; figure 1A), from SCD (HR 2.5 (1.6 to 3.9); p<0.001; figure 1B), from progressive heart failure (HR 1.6 (1.1 to 2.3); p=0.02) and from non-cardiovascular causes (HR 1.5 (1.1 to 2.2); p=0.027) than those not fulfilling TA314 criteria.

### Guideline performance according to clinical context

**Figure 2:**
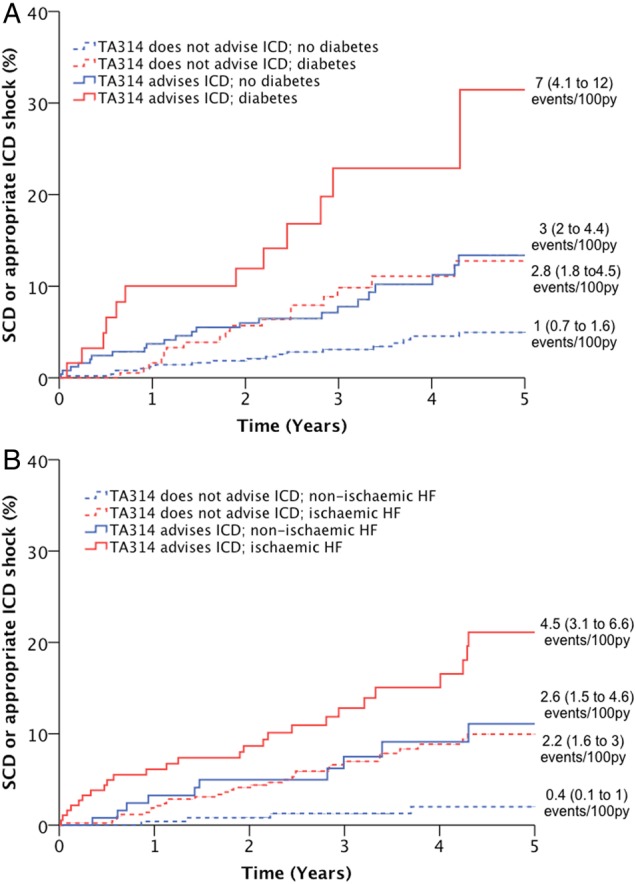
Performance of standard TA314 criteria in patients with diabetes or ischaemic aetiology. Kaplan–Meier curves demonstrating that within populations stratified by (A) diabetes or (B) ischaemic aetiology, TA314 identifies subgroups at increased relative risk of sudden death or appropriate ICD shock (p<0.05 for all within stratum comparisons of TA314). However, absolute event rates in subgroups of patients meeting TA314 criteria were similar to those in subgroups not meeting TA314 criteria, depending on the presence of diabetes or ischaemic aetiology. HF, heart failure; ICD, implantable cardioverter-defibrillator; SCD, sudden cardiac death.

Univariate predictors of SCD were sought to define clinical contexts where guideline performance may vary (table 2). This indicated that diabetes and ischaemic aetiology are also important predictors of risk, and these observations persisted in a multivariate analysis including diabetes, ischaemic aetiology and NICE TA314 status (table 3). Moreover, there was no interaction between TA314 and diabetes or ischaemic aetiology, indicating a similarly adverse impact of these comorbidities independent of TA314 status. Furthermore, study recruitment era (first vs second half of study recruits) did not interact with TA314, suggesting no change in performance over time. We then produced Kaplan–Meier curves of SCD risk in patients with or without diabetes (figure 2A) or ischaemic aetiology (figure 2B), according to NICE TA314 recommendation status. These clearly illustrate that although the guidelines define groups with increased *relative* risk, the *absolute* risk of SCD remains highly influenced by clinical context. For example, event rates in patients with diabetes not meeting TA314 criteria were similar to patients without diabetes who met TA314 criteria.

**Table 2:** Univariate predictors of SCD or appropriate ICD shock

		95% CI of HR			
Variable	HR	Low	High	p Value	Wald
Age (per year)	1.02	1	1.04	0.045	4
Male sex	1.92	1.06	3.47	0.032	4.6
LVEF (per %)	0.967	0.945	0.99	0.005	7.9
QRS interval (per ms)	1.011	1.004	1.018	0.002	9.9
eGFR (per mL/kg/1.73 m^2^)	0.989	0.976	1.002	0.086	2.9
Haemoglobin (per g/dL)	0.987	0.974	0.999	0.045	4
NYHA class (vs I)				0.33	3.4
II	1.7	0.89	3.24		
III	1.87	0.94	3.69		
IV	1.5	0.19	11.5		
Ischaemic aetiology	2.68	1.55	4.64	<0.001	12.4
Diabetes	2.36	1.51	3.7	<0.001	14.1
TA314 recommends ICD	2.53	1.62	3.94	<0.001	16.8

eGFR, estimated glomerular filtration rate; ICD, implantable cardioverter-defibrillator; LVEF, left ventricular ejection fraction; NYHA, New York Heart Association; SCD, sudden cardiac death.

**Table 3:** Multivariate predictors of SCD or appropriate ICD shock

		95% CI of HR			
Variable	HR	Low	High	p Value	Wald
TA314 recommends ICD	2.76	1.77	4.31	<0.001	19.9
Diabetes	2.17	1.37	3.45	0.001	10.8
Ischaemic aetiology	2.43	1.39	4.26	0.002	9.7

ICD, implantable cardioverter-defibrillator; SCD, sudden cardiac death.

### Sensitivity analyses

**Figure 3:**
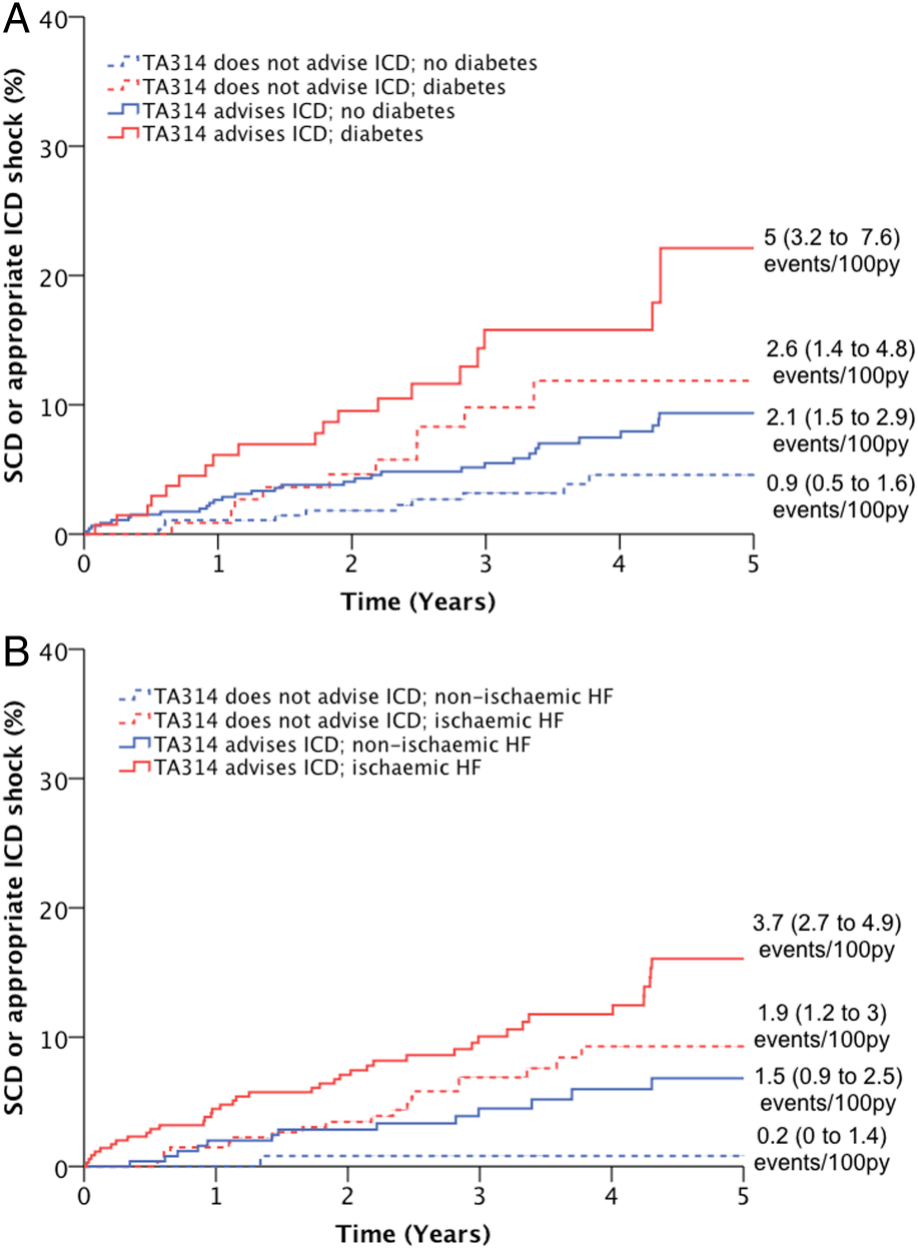
Performance of extended TA314 criteria in patients with diabetes or ischaemic aetiology. Kaplan–Meier curves demonstrating that within populations stratified by (A) diabetes or (B) ischaemic aetiology, TA314 identifies subgroups at increased relative risk of sudden death or appropriate ICD shock (p<0.05 for all within stratum comparisons of TA314). However, absolute event rates in subgroups of patients meeting TA314 criteria were similar to those in subgroups not meeting TA314 criteria, depending on the presence of diabetes or ischaemic aetiology. HF, heart failure; ICD, implantable cardioverter-defibrillator; SCD, sudden cardiac death.

TA314 supports the provision of ICDs to patients with QRS duration <120 ms who are deemed to be at high risk of SCD, although provides no specific guidance regarding how to make this assessment. We, therefore, conducted a sensitivity analysis of extended TA314 criteria, in which ICD candidacy required only LVEF ≤35% and NYHA class I–III symptoms (59.6% of patients). Patients meeting extended TA314 criteria were no longer at increased risk of all-cause mortality (HR 1.2 (1.0 to 1.5); p=0.13), but remained at greater risk of SCD (HR 2.0 (1.2 to 3.4); p=0.007). In multivariate analysis identical to that presented in table 3, extended TA314 criteria, diabetes and ischaemic aetiology all remained independently associated with the risk of SCD. The impact of diabetes or ischaemic aetiology on extended TA314 criteria performance is further illustrated in figure 3, reproducing our observations regarding standard TA314 criteria in figure 2.

**Figure 4:**
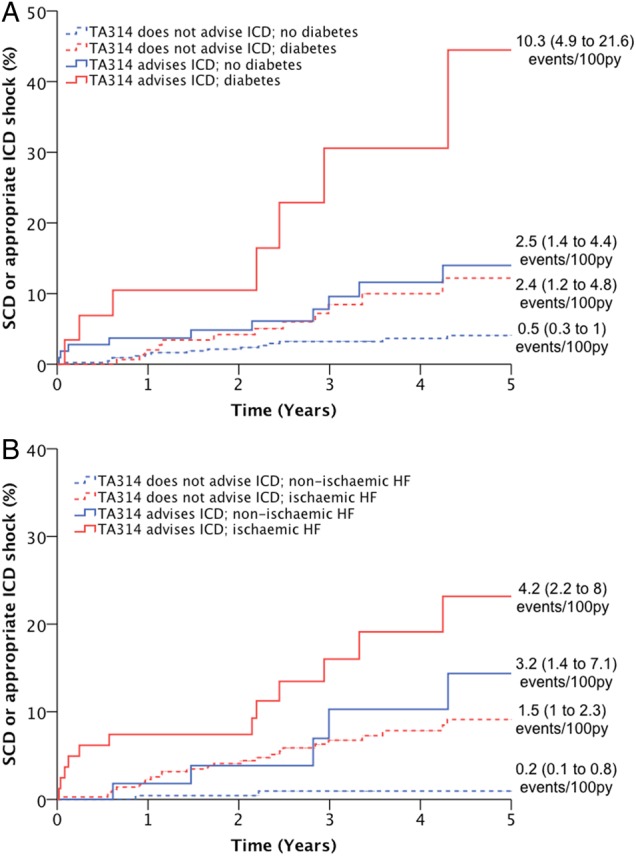
Performance of standard TA314 criteria in patients with diabetes or ischaemic aetiology, after excluding patients with prior device therapy. Kaplan–Meier curves demonstrating that within populations stratified by (A) diabetes or (B) ischaemic aetiology, TA314 identifies subgroups at increased relative risk of sudden death or appropriate ICD shock (p<0.05 for all within stratum comparisons of TA314). However, absolute event rates in subgroups of patients meeting TA314 criteria were similar to those in subgroups not meeting TA314 criteria, depending on the presence of diabetes or ischaemic aetiology. HF, heart failure; ICD, implantable cardioverter-defibrillator; SCD, sudden cardiac death.

As patients with current device therapy may experience a differential risk of SCD, we also conducted a sensitivity analysis of standard TA314 criteria performance by excluding all patients with device therapy (n=316). In this scenario, TA314 was associated with risk of all-cause mortality (HR 2.3 (1.7 to 3.0); p<0.001) and risk of SCD (HR 3.9 (2.0 to 7.6); p<0.001). In multivariate analysis identical to that presented in table 3, standard TA314 criteria, diabetes and ischaemic aetiology all remained independently associated with the risk of SCD. The impact of diabetes or ischaemic aetiology on standard TA314 criteria performance is further illustrated in figure 4, reproducing our observations regarding standard TA314 criteria in figure 2.

## Discussion

This study of >1000 patients with heart failure examines the ability of the NICE TA314 guidelines to identify patients at risk of SCD. The most important findings are as follows: (1) between 30% and 60% of unselected patients with heart failure are now eligible to receive an ICD, depending on the attribution of high-risk status to patients with narrow QRS interval; (2) overall risk of SCD in patients with heart failure due to reduced LVEF is approximately 2% per annum, making risk stratification for ICD implantation a clinical priority; (3) patients fulfilling the TA314 criteria for ICD implantation are at increased risk of SCD, compared with those not fulfilling the criteria and (4) the performance of TA314 in defining high absolute risk of SCD is significantly influenced by clinical context. This means that some patient subgroups meeting TA314 criteria for ICD implantation experience similar absolute risk of SCD to other subgroups that do not meet TA314 criteria for ICD implantation.

The use of ICD in patients with heart failure due to reduced LVEF is now a mainstay of treatment worldwide with >5000 devices implanted in the UK during 2012, a rise of >10% from 2009.^19^ These devices are well tolerated and, after initial concerns, it has been shown that after implantation patients do not suffer from deterioration in their quality of life.^20^ Guidelines from the American Heart Association, American College of Cardiologists and the Heart Rhythm Society recommend ICD therapy for patients with LVEF ≤35% due to myocardial infarction who are at least 40 days postmyocardial infarction and are in NYHA functional class II or III, or in patients who have an EF ≤30% and are in NYHA class I.^21^ The European Society of Cardiology guidelines state that patients should receive an ICD if in NYHA class II–III with an LVEF ≤35% in spite of 3 months of optimal pharmacological therapy and expected to survive for 1 year with good functional status.^22^

The UK NICE guidelines in 2006 recommended ICD implantation in patients who had sustained an myocardial infarction (MI) at least 40 days previously with an LVEF ≤30% with QRS duration on 12-lead ECG ≥120 ms.^23^ It was also advised that patients with an LVEF ≤35% and non-sustained ventricular tachycardia on ambulatory ECG and a positive electrophysiological test should also receive an ICD. The recently updated NICE TA314 guidelines now base stratification of patients with LVEF ≤35% on QRS duration and NYHA functional class, with ICD implantation being recommended in patients in NYHA class I–III and QRS duration >120 ms. It is also recommended that where patients meet LVEF and NYHA criteria, but have QRS <120 ms, they should receive an ICD if there is high risk of sudden death; no guidance was provided on how to identify such patients. Importantly, the updated guidelines no longer suggest accounting for ischaemic aetiology during the decision-making process. This reflects expert evidence the guideline panel received, stating that ‘the aetiology (ischaemic/non-ischaemic) does not influence the effectiveness of ICD therapy’.^14^

Periodic updates of NICE guidelines have the potential to maintain ‘best practice’ and improve clinical outcomes across the UK, sometimes by advocating major changes in clinical decision-making. These changes may also have substantial economic and personal impact, and must carefully consider the threshold between overtreatment and undertreatment. Notably, following TA314 guidance would have significantly increased ICD use in our ‘historic’ cohort. ICD guidelines, including TA314, have been based on evidence provided by clinical trials, which recruit patients who are often very different to those presenting in routine clinical practice.^24^ Additional real-world data regarding the performance of these guidelines are therefore essential, since they have the potential to reassure healthcare providers and patients that ICD therapy is appropriate. In the present analysis, we have demonstrated that NICE TA314 guidelines identify a group of patients at significantly increased relative risk of sudden death. However, absolute risk prediction is also crucial from a clinical and cost-effectiveness perspective, and our data suggest that in this regard the performance of TA314 is highly context specific. We recognise the significant effort involved in formulating TA314, including the conduct of an individual patient network meta-analysis that required access to data from multiple industry sponsors. Some of these important and thorough analyses have recently been published,^25^ and support the notion that the benefit of ICD therapy differs between patient subgroups. We hope that future guidelines will be able to build on these data, and our analyses, to further refine risk stratification by considering other SCD risk factors, and ensure calibration to contemporary real-life event rates.

### Study limitations

This study is a retrospective analysis, although of a prospectively performed cohort study, and this must be borne in mind when interpreting the results. In particular, retrospective analysis makes it difficult to predict how competing causes of death would change if ICD usage followed TA314 guidance. A second limitation is the use of appropriate shocks as a surrogate for sudden death as the underlying arrhythmia may not have been subsequently fatal. While this may serve to overestimate the capacity of TA314 to predict risk of SCD, it is reassuring to note our sensitivity analysis excluding patients with device therapy reached broadly similar conclusions. Our interpretation of TA314 guidance for patients with narrow QRS duration was conservative, although our sensitivity analysis including patients with narrow QRS arrived at similar conclusions.

## Conclusion

The recently updated NICE guidelines on ICD implantation can identify patients with reduced LVEF at increased relative risk of sudden death. Clinicians should also consider clinical context and the absolute risk of SCD when advising patients about the potential risks and benefits of ICD therapy.

**Key messages**

**What is already known on this subject?**

- Chronic heart failure is associated with increased risk of sudden death, which can be mitigated by implantable cardioverter-defibrillators (ICDs).
- The National Institute for Health and Care Excellence suggest that ICD use is cost-effective in selected patients, outlined in the recently updated TA314 guidance.

**What might this study add?**

- TA314 identifies populations at increased risk of sudden death.
- The performance of TA314 is sensitive to patient context, including comorbid diabetes and ischaemic heart disease.

**How might this impact on clinical practice?**

- When risk-stratifying individuals according to TA314 guidance on implantable cardioverter-defibrillator use, clinicians should also consider comorbid diabetes and ischaemic heart disease.
- Future updates to TA314 should consider refining risk-stratification approaches to minimise both preventable sudden death and overtreatment.
